# The Matron's Department

**Published:** 1905-10-21

**Authors:** 


### HOSPITAL ADMINISTRATION.

### CONSTRUCTION AND ECONOMICS.

### THE MATRON'S DEPARTMENT.

#### X. THE COMMISSARIAT.

The part which the matron is called on to play with regard to victualling the institution necessarily varies in accordance with its size. In the case of small households it may fall to the matron to supervise the buying and storing of the provisions, as well as their preparation and distribution. It is, however, more usual for the secretary or steward to arrange contracts for provisions under the sanction of the governors and the responsibilities of the matron do not in that case begin until the goods are delivered on the premises.[1]

It should be an invariable rule in every household to have each article weighed or measured and carefully inspected on delivery, and no pressure of work should be allowed to interfere with this duty. It greatly facilitates matters when a small room can be set apart for the reception of all goods as they come in, but if this cannot be managed some part of the ordinary store-room must be reserved for the purpose. The housekeeper should herself superintend the inspection whenever it is possible, and even with such operations as weighing bread and meat, which recur daily and must be done immediately, she should make a point of being present occasionally, and should receive a daily report from the officer in charge as to the result. Where there is constant cause for complaint, as happens in many parts of the country with regard to the quality of the bread, the matron should obtain powers to return the goods to the contractor and purchase elsewhere; there is usually in every contract a clause to this effect which is too often allowed to become a dead letter.

The allowance system is now generally discredited, and the housekeeper must provide collectively, instead of individually, for the needs of the household. In order to do so to the best possible advantage, the procedure should be as follows:—

The Sister of each ward will furnish a report every afternoon of the requirements for her ward on the following day, noting on diet sheets provided for the purpose the diets and extras ordered for her patients. The housekeeper will then combine these into a form which will show the day's diet and the quantities required for all the patients in the hospital. She must next, after due consultation with the cook and inspection of what is left over, make out the bill of fare for the various meals required by nursing staff, medical officers, and servants. Lastly, she will combine these requirements into one with those of the patients in her Daily Requisition Sheet in which the quantities required under every heading are noted. We give here an example.

[1] We refer our readers for a more detailed account of the best methods of hospital housekeeping to "Hospital Expenditure: The Commissiarat." (Scientific Press.)

*Housekeeper's Daily Requisition Sheet.*

| — | | Patients | Medical Officers | Nurses | House |
|---|---|---|---|---|---|
| Meat .. .. | 1. Beef .. .. | | | | |
| | 2. Mutton .. .. | | | | |
| | 3. Shin for beef-tea, etc. .. .. | | | | |
| Fish .. .. | Ordinary supplies | | | | |
| Poultry .. | 1. Fowls .. .. | | | | |
| | 2. Rabbits, etc. .. | | | | |
| Bread, Flour, etc. .. | 1. Bread .. .. | | | | |
| | 2. Flour .. .. | | | | |
| | 3. Oatmeal .. .. | | | | |
| Vegetables and Fruit.. | 1. Potatoes .. .. | | | | |
| | 2. Other vegetables.. | | | | |
| | 3. Fruit .. .. | | | | |
| Milk and Cream .. | 1. Milk .. .. | | | | |
| | 2. Cream .. .. | | | | |
| Cheesemongery | 1. Butter .. .. | | | | |
| | 2. Cheese .. .. | | | | |
| | 3. Ham and bacon .. | | | | |
| Eggs.. .. | 1. Cooking .. .. | | | | |
| | 2. New-laid .. .. | | | | |
| Grocery .. | 1. Tea .. .. .. | | | | |
| | 2. Coffee .. .. | | | | |
| | 3. Cocoa .. .. | | | | |
| | 4. Jams, etc... .. | | | | |
| Aerated Waters .. | Aerated Waters .. | | | | |
| Alcohol .. | Various headings .. | | | | |

It is of the utmost importance that the preparation of this requisition sheet should not be omitted from among the daily duties of the housekeeper, be the numbers large or small for whom she has to cater. Having ascertained the requirements of the household, the housekeeper will then be able to give signed orders for the perishable provisions which come in day by day, and will herself issue such stores as tea, sugar, bacon, cheese, etc. A good deal of skill is required in weighing out stores, and the custom of measuring by spoonfuls is answerable for much dissatisfaction and waste. If it is necessary to employ an assistant in this work, she should be trained to rigid accuracy. Each transaction should be noted in pencil on a tiny square of paper, stabbed on to a labelled file adjoining each receptacle, so that, for instance, a rapid calculation may be made at any time of the number of pounds of tea which have been used to date. These memoranda will serve as a check on careless distribution of provisions, which results in much needless expenditure.

The matron is not herself responsible for the diets provided for the patients; but she is responsible for the condition in which these diets are served, and it is very necessary that she should personally superintend the manner in which the patients are fed. She should visit the wards from time to time during meal times, and without any undue fussiness she should take notice whether the food is left on the plates, whether it is served cold or is in any way unappetising, and should consult with the sister of the ward with a view to making alterations if necessary. In hospitals unprovided with modern apparatus for keeping the dinners hot on the way from the kitchen there is often great difficulty in this direction. But if the matron is known to lay stress upon the patients getting their meals served nicely the probability is that these difficulties will be found possible to overcome. The reputation of a hospital in small country towns is often affected by the quality of the meals more than is generally supposed, and it is only fair to the patients to take some pains to see that they receive all that is ordered for them served to the best advantage.

The arrangement of meals for the hospital household is exceedingly complicated, and the preliminary step of settling the time-table is by no means an easy one. We quote an example from a medium-sized hospital where the nursing staff numbers about 50.

*Meals Served Daily.*

| Hour. | For Whom. | Approximate Number. |
|---|---|---|
| 6.40 a.m. | First nurses' breakfast .. .. .. .. | 26 |
| 7.40 a.m. | Second sisters' breakfast, quarters' maids breakfast | 10, 4 |
| 7.30 a.m. | Servants' hall breakfast .. .. .. .. | 10 |
| 8 a.m. | Porters' mess .. .. .. .. .. | 4 |
| | Steward's breakfast, housekeeper's breakfast .. | 2 |
| 9 a.m. | First dinner (night nurses) .. .. .. | 12 |
| 11.30 a.m. | Patients' dinners .. .. .. .. .. | About 110 |
| 12.0 | Second dinner (Sisters and nurses) .. .. | 20 |
| | First men's mess-room dinner .. .. .. | 9 |
| | Dispensary porter, gate boy .. .. .. | 2 |
| 12.30 p.m. | Doctors' luncheon .. .. .. .. .. | 6 |
| 1 p.m. | Third dinner (nurses), steward's mess .. .. | 22, 4 |
| | Trays to secretary and dispenser, and sundry rations | 11 |
| 1.15 p.m. | Servants' hall .. .. .. .. .. | 9 |
| 4 to 5 p.m. | Teas for secretary, office, mess-room, and servants' hall | 25 |
| 7 p.m. | Doctors' dinners .. .. .. .. .. | 5 |
| 8 p.m. | First nurses' supper .. .. .. .. | 26 |
| 8.40 p.m. | Second nurses' supper, quarters' maids supper | 23, 4 |
| 8 p.m. | Servants' hall, porters' suppers .. .. .. | 15 |
| | Total .. .. .. .. | 359 |

This table includes only the meals supplied from the kitchen. Teas for the patients and nurses and breakfast for the patients supplied from the ward kitchens do not fall under this heading. But these must of course be included in the housekeeper's requisition-sheet, so that the necessary supplies may be issued.

There is very just complaint in hospitals against the constraint and discomfort which too commonly reigns during meals. It is entirely within the matron's power to make these gatherings a time of refreshment both to mind and body, but to this end she must take a little trouble. The usual practice of having one long bare table is bad. It checks conversation in all but inveterate talkers, and those who must come in late or leave early lend an air of hurry and makeshift to the meal. The tables should be separated and laid for not more than eight or ten. Where the numbers are not large, two tables with six at each is a far more sociable arrangement than one with twelve, and the waiting is rather facilitated than otherwise. The matron presides at the sisters' table and appoints a sister to preside at each of the others. A separate table is provided for those whose meal-time may not quite coincide with the rest, and hot-water plates with covers are served for those who are delayed by their duties.

It is of the greatest importance that the food provided should be light and easily digested. Articles of diet which make a great strain upon the digestive organs should be either altogether avoided, or should be served with an alternative. Such dishes, for instance, as salt beef and roast pork, are not suitable even for people with good digestions, when they must leave the table to go back at once to hard work. The matron may possibly not be able to have her own way altogether as regards the supplies. She may have to make the best of hard beef and tough mutton, but she will find a way out of the difficulty if she consults a good cookery-book. Beef, which is almost uneatable when baked till it is dry and hard in the usual way, can be made into a delicious dish when braised, and the toughest mutton will make excellent haricot. It is not monotony which is so much to be dreaded in the hospital bill of fare as indigestion. But monotony too can be avoided if the matron will think out six or eight forms of menu and bear seasonal changes in view. Variety is too dearly purchased, however, when it is attained at the sacrifice of food value, and a slice of cold meat, however uninteresting, makes a more satisfactory supper for a busy woman than tinned salmon or potted shrimps. Too much stress cannot be laid on the importance of good carving; and since no matron is possessed of all the gifts, she will do well, if she is not herself a proficient in this art, to delegate it to a skilled hand among the staff. It is better that the carving should be done in the room. It is very usual indeed to hear nurses complain that they cannot tell what they are eating, as there is never any difference between the beef and mutton, and the same remark may often be heard at hotels and restaurants, showing how large a part imagination plays in the sense of taste. Hence the advantage to the appetite of actually seeing the joint carved. It is not our purpose to suggest in this place any particular form of dietary. Luxuries are not needed, although plenty of sweet things—treacle, jam, and marmalade—are a necessity to persons engaged in manual labour. But if the staff is to be maintained in the best possible condition as regards their work the matron must take pains to see that the food provided is well cooked, well served, well carved, and eaten in an atmosphere of serenity and order.

